# Vinculin is required to maintain glomerular barrier integrity

**DOI:** 10.1016/j.kint.2017.09.021

**Published:** 2018-03

**Authors:** Franziska Lausecker, Xuefei Tian, Kazunori Inoue, Zhen Wang, Christopher E. Pedigo, Hossam Hassan, Chang Liu, Margaret Zimmer, Stephanie Jinno, Abby L. Huckle, Hellyeh Hamidi, Robert S. Ross, Roy Zent, Christoph Ballestrem, Rachel Lennon, Shuta Ishibe

**Affiliations:** 1 Wellcome Trust Centre for Cell-Matrix Research, Faculty of Biology, Medicine and Health, University of Manchester, Manchester, UK; 2 Department of Internal Medicine, Yale University School of Medicine, New Haven, Connecticut, USA; 3 Department of Medicine/Cardiology, University of California, San Diego, School of Medicine, La Jolla, California, USA and Veterans Affairs San Diego Healthcare System, San Diego, California, USA; 4 Department of Medicine, Vanderbilt University Medical Center, Nashville, Tennessee, USA; 5 Veterans Affairs Hospital, Nashville, Tennessee, USA; 6 School of Biology, Faculty of Biology, Medicine and Health, University of Medicine, Manchester Academic Health Science Centre, UK

**Keywords:** cell adhesion, glomerular disease, glomerular filtration barrier, podocyte, vinculin

## Abstract

Cell-matrix interactions and podocyte intercellular junctions are key for maintaining the glomerular filtration barrier. Vinculin, a cytoplasmic protein, couples actin filaments to integrin-mediated cell-matrix adhesions and to cadherin-based intercellular junctions. Here, we examined the role of vinculin in podocytes by the generation of a podocyte-specific knockout mouse. Mice lacking podocyte vinculin had increased albuminuria and foot process effacement following injury *in vivo*. Analysis of primary podocytes isolated from the mutant mice revealed defects in cell protrusions, altered focal adhesion size and signaling, as well as impaired cell migration. Furthermore, we found a marked mislocalization of the intercellular junction protein zonula occludens-1. In kidney sections from patients with focal segmental glomerulosclerosis, minimal change disease and membranous nephropathy, we observed dramatic differences in the expression levels and localization of vinculin. Thus, our results suggest that vinculin is necessary to maintain the integrity of the glomerular filtration barrier by modulating podocyte foot processes and stabilizing intercellular junctions.

The glomerular filtration barrier is composed of highly specialized visceral epithelial cells (podocytes), the glomerular basement membrane (GBM), and fenestrated endothelial cells. The barrier prevents macromolecules in the blood from traversing into the urine, and defects in the barrier result in albuminuria and nephrotic syndrome. Recent evidence has demonstrated the importance of cell–matrix interactions in podocytes.^1^ These interactions are mediated by cell surface integrin receptors binding to ligands in the GBM. Human and mouse genetic studies have shown that ablation or mutations in α3β1 integrin,^2^, ^3^, ^4^ along with its ligand, β2 laminin,^5^ result in severe proteinuria, and that genetic ablation of integrin binding proteins—integrin linked kinase (ILK),^6^ talin1,^7^ and tetraspanin CD151^8^—leads to phenocopying of integrin loss, emphasizing the importance of cell–matrix interactions for barrier function. Furthermore, the integrity of the filtration barrier is also governed by the podocyte slit diaphragm, a modified tight junction that connects interdigitating foot processes, and serves as the terminal barrier in the glomerulus.^9^ Patients harboring genetic mutations in slit diaphragm proteins, such as nephrin, FAT1, or podocin, present with severe, steroid-resistant nephrotic syndrome.^10^

Vinculin is an adapter protein that localizes at cell–matrix adhesions (also known as focal adhesions [FAs])^11^ and at cell–cell junctions.^12^ It comprises an N-terminal head domain, a proline-rich linker, and a C-terminal tail domain. The head domain binds to talin at cell–matrix adhesions and predominantly to α-catenin at cell–cell junctions.^13^ The tail domain has been shown to bind paxillin, F-actin, and phosphatidylinositol-4,5-bisphosphate.^14^, ^15^, ^16^ Vinculin has a well characterized function of stabilizing cell–matrix adhesions by orchestrating the recruitment and release of other cell–matrix adhesion proteins, thereby controlling the strength of adhesion binding to the extracellular matrix (ECM).^17^, ^18^, ^19^ Vinculin also has been shown to regulate cell migration,^20^ which is determined by the adhesive state of cells mediated by FAs and their dynamic turnover.^21^ Although it is clear that vinculin modulates cell adhesion, its role in the glomerular filtration barrier is unclear.

Aside from its function in cell–matrix adhesions, vinculin is an important regulator of cell–cell junctions.^22^, ^23^ Mice lacking vinculin, specifically in cardiomyocytes, die suddenly of ventricular arrhythmias due to disruption of gap junctions containing intercalated disks at cell–cell junctions. If they survive this early vulnerable period, such mice later succumb to heart failure from a dilated cardiomyopathy, caused by disruption of both cell–matrix and cell–cell junctions.^24^, ^25^ Vinculin also is known to interact with zonula occludens (ZO)-1 in the cardiac myocyte; with vinculin deficiency, an associated reduction in ZO-1 occurs.^25^ The importance of ZO-1 in the function of podocytes is best illustrated by *Tjp1* (or ZO-1) knockout (KO) studies, as mice lacking this gene develop severe proteinuria.^26^

Given that vinculin (Vcl) plays a pivotal role in regulating cell–matrix and cell–cell adhesion, and that it binds ZO-1, we hypothesized that it has a critical role in glomerular filtration. To circumvent the embryonic lethality seen in the global *Vcl* KO mouse,^21^ we generated a podocyte-specific *Vcl* KO mouse using the *Podocin-Cre* and *Vcl* ^flox/flox^ system (Pod-*Vcl-*KO). These mice were generally healthy, with only mild proteinuria. However, Pod-*Vcl-*KO mice were more susceptible to proteinuria and foot process effacement after injury. Isolated Pod-*Vcl*-KO podocytes demonstrated an altered FA size, increased phosphorylation levels of FA protein FAK, and increased cell migration. Additionally, marked mislocalization of the junctional protein ZO-1 occurred after injury. Finally, in kidney biopsies from patients with focal segmental glomerulosclerosis (FSGS), minimal change disease (MCD), and membranous nephropathy (MN), marked differences were found in vinculin localization that correlated with the level of nephrin localization in capillary loops. Our findings indicate that vinculin plays a critical role in maintaining the glomerular barrier by regulating podocyte foot process morphology and localizing ZO-1 at the plasma membrane.

## Results

### Generation of the podocyte-specific *Vcl*-KO mouse

**Figure 1:**
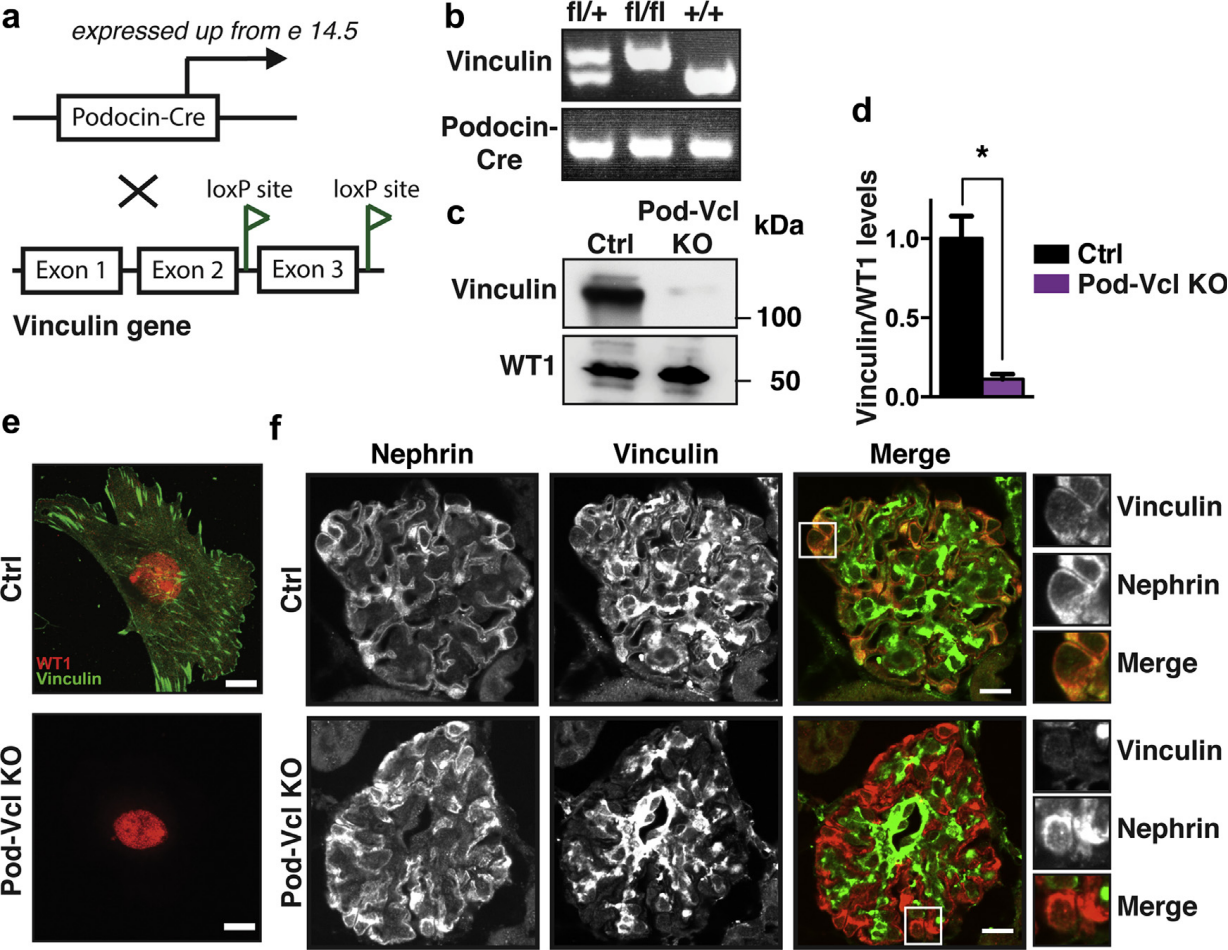
**Generation of podocyte-specific *Vcl-KO* mice.** Schematic demonstrating the breeding of the *Podocin*-*Cre* mice with *Vcl*^flow/flow^ mice to generate the podocyte-specific *Vcl-KO* mice (Pod-*Vcl*-KO; **a**). Identification of *Vcl*^*fl/fl*^ and *Podocin-Cre* by tail genotyping (**b**). Representative vinculin protein level in purified control (Ctrl) podocytes and loss of immunoreactivity of vinculin in podocytes harvested from Pod-*Vcl*-KO mice as detected by Western blotting; n = 3 independent experiments (**c**). Quantification of vinculin protein levels relative to WT1 protein levels: N = 5; *P < 0.001 (**d**). Localization of vinculin and wild type (WT)1 in control and Pod-*Vcl*-KO primary podocytes by immunofluorescence (bar = 10 μm; **e**). Double immunofluorescence of nephrin and vinculin on kidney sections of the indicated genotypes (**f**). In the merged image, vinculin is shown in green; nephrin is shown in the red channel (bar = 10 μm). To optimize viewing of this image, please see the online version of this article at www.kidney-international.org.

To study the role of vinculin in podocytes, we generated a podocyte-specific vinculin KO mouse (Pod-*Vcl*-KO) using the Cre–Lox system based on podocin cre, which is expressed at E14^27^, and vinculin floxed transgenic mice^24^ (Figure 1a). The Pod-*Vcl*-KO mice were born in the normal Mendelian frequency as identified by tail genotyping (Figure 1b). Isolated, enriched primary podocytes, harvested from the Pod-*Vcl*-KO mouse and identified by the podocyte-specific marker Wilms Tumor-1 (WT1), showed reduced vinculin expression, as determined by western blot analysis (Figure 1c; quantified in Figure 1d) and immunocytochemistry (Figure 1e). Kidneys from Pod-*Vcl*-KO mice and control littermates (termed control mice) were immunostained for vinculin and the podocyte-specific marker nephrin, which confirmed that the observed loss of vinculin was podocyte specific, as co-localization of the markers was lost in the Pod-*Vcl*-KO (Figure 1f). In summary these data demonstrate the successful elimination of vinculin from podocytes in the Pod-*Vcl*-KO mouse.

### Vinculin is required for podocyte integrity after injury

**Figure 2:**
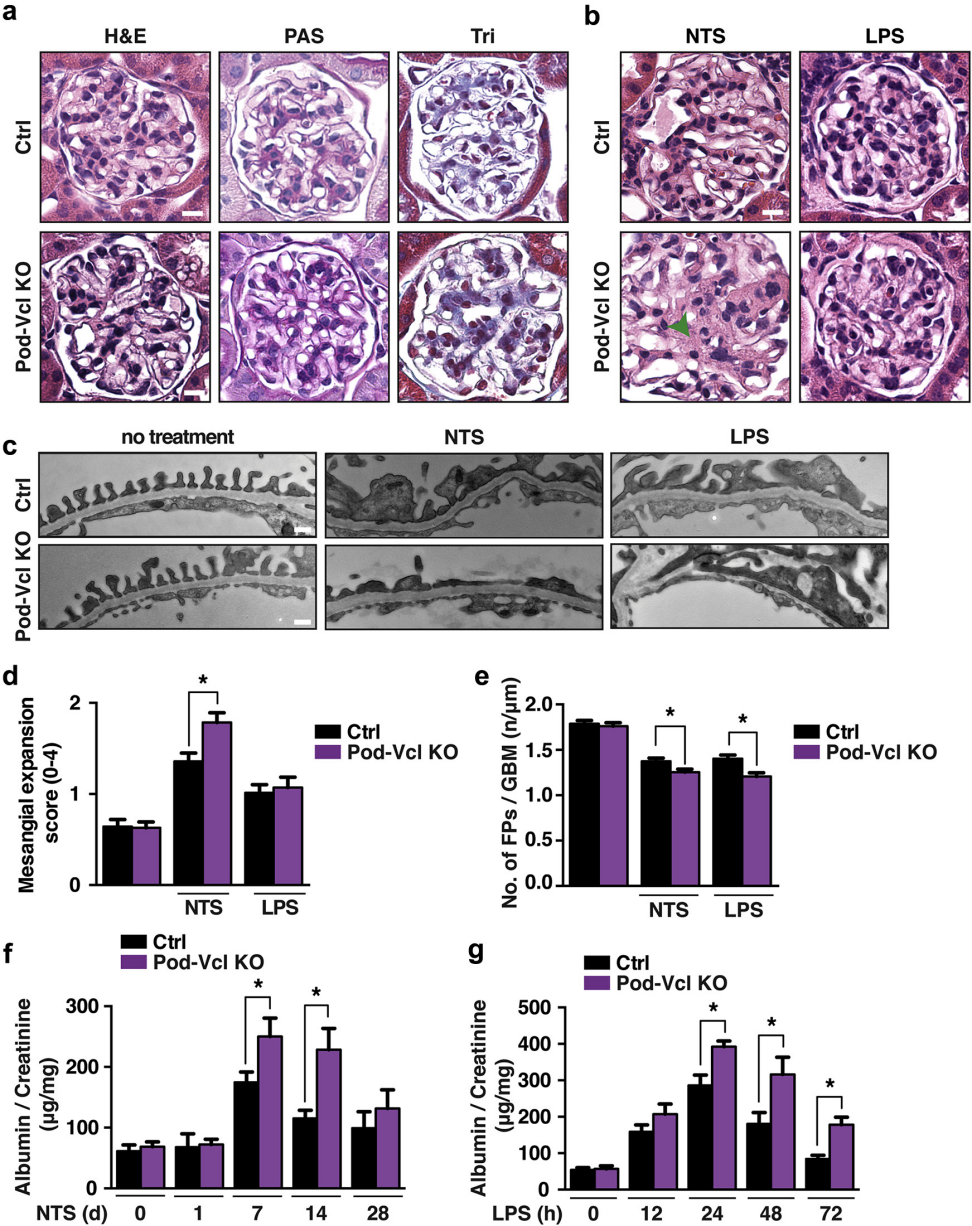
**Loss of *Vcl* in podocytes results in worsened albuminuria and foot process effacement.** Representative light microscopy images of glomeruli from control and Pod-*Vcl-*KO mice at age 8 weeks, stained with hematoxylin and eosin (H&E), periodic acid–Schiff (PAS), and Trichrome (Tri), which reveal no significant differences (bar = 20 μm; **a**). Representative images of control (Ctrl) and Pod-*Vcl*-KO mice at age 8 weeks after nephrotoxic serum (NTS) injection at 7 days and lipopolysaccharide (LPS) treatment after 24 hours (bar = 20 μm; **b**). Transmission electron micrographs revealing foot process effacement in Ctrl and *Pod-Vcl-KO* mice after LPS treatment (24 hours) and NTS injection (7 days) (bar = 150 nm; **c**). Quantification of (**b**); mesangial expansion was assessed with a score from 0 to 4, with 0 representing no detectable mesanigal expansion, and 4 being severe, by blinded pathologist. A total of 15–20 glomeruli were analyzed from n = 4 mice; *P < 0.01 (**d**). Quantification of (**c**), the number of foot processes per micrometer of glomerular basement membrane in the Ctrl and Pod-*Vcl*-KO mice after NTS or LPS injection. N = 3 mice were used at each time point. *P < 0.001 (**e**). Quantification of urinary albumin normalized to creatinine at 0, 1, 7, 14, and 28 days after NTS. N = 10 mice at each time point; *P < 0.001. Error bars show SEM (**f**). Quantification of urinary albumin normalized to creatinine at 0, 12, 24, 48, and 72 hours after LPS treatment. N = 10 mice at each time point; *P < 0.001. Error bars show SEM (**g**). To optimize viewing of this image, please see the online version of this article at www.kidney-international.org.

**Figure S1:**
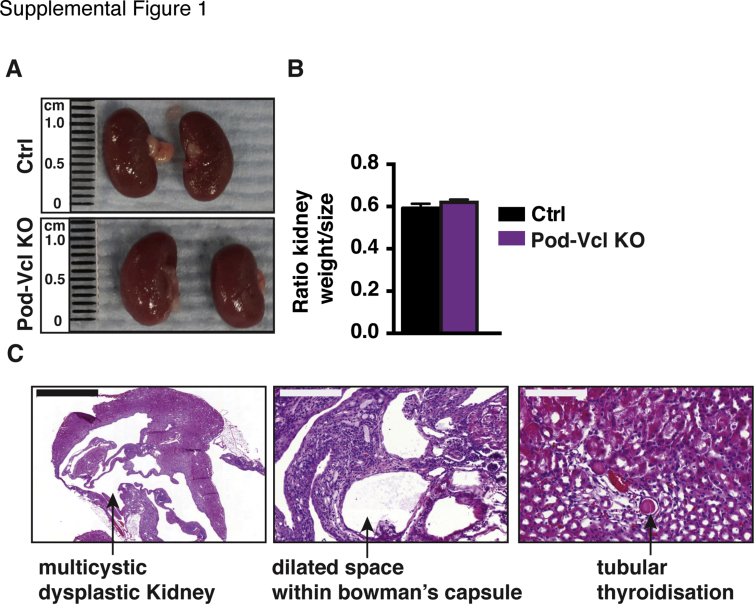
Kidney abnormalities are detected in mice of advanced age. Most kidneys from Vcl KO and control (Ctrl) mice did not a reveal a morphologic difference (**A**). Kidney weight (g) was analyzed relative to kidney size (cm) (**B**). Hematoxylin and eosin staining of Pod-*Vcl*-KO mice shows sporadic cystic abnormalities. Left image shows multiple cysts within the kidney of a *Pod-Vcl-KO* mouse. The Bar = 2 mm. The middle image shows cystic abnormalities within the glomerular Bowman’s capsule. Bar = 200 μm. The right image shows crystallization of protein in tubular structures. Bar = 100 μm (**C**).

As evidence of the rigorous and reproducible results from this animal model, Pod-*Vcl*-KO mice were generated independently in our two laboratories, and most (approximately 90%) of the mice were normal (studied to age 10 months). No differences were observed between Pod-*Vcl*-KO and control mice, with respect to kidney morphometry (weight or size) (Supplementary Figure 1A; quantified in Supplementary Figure 1B), nor was there evidence of alteration in serum creatinine levels in the Pod-*Vcl*-KO (data not shown). Renal histology demonstrated normal glomerular architecture, with no evidence of fibrosis compared with age-matched control mice (Figure 2a). A total of 38% of aged Pod-*Vcl*-KO mice (age ≥9 months) kidneys had large glomerular cysts (Supplementary Figure 1C). These cysts were not observed in any of the younger Pod-*Vcl*-KO mice.

Given that basal findings of the Pod-*Vcl-*KO mice showed only minor abnormalities, we next challenged these mice by administering rabbit anti-mouse GBM (nephrotoxic serum [NTS]). At 7 days after NTS injection, the Pod-*Vcl*-KO mice exhibited glomerular damage characterized by increased mesangial matrix accumulation (Figure 2b). Transmission electron microscopy (TEM) revealed more foot process effacement in the Pod-*Vcl*-KO mice treated with NTS, compared with control mice (Figure 2c). Quantification of mesangial matrix accumulation and foot process effacement reveals significant differences between NTS-treated Pod-*Vcl*-KO mice and control mice treated with NTS (Figure 2d and e). We also challenged the mice with i.p. lipopolysaccharide (LPS), which is considered to be an animal model for minimal change disease, and we observed no histologic differences using light microscopy (Figure 2b; quantified in Figure 2d). Ultrastructural examination by TEM showed an increase in podocyte injury, as evidenced by foot process effacement in the Pod-*Vcl*-KO compared with control mice (Figure 2c; quantified in Figure 2e). Lastly, functional analysis demonstrated that after either NTS- or LPS-induced glomerular injury, the Pod-*Vcl*-KO mice had significantly more albuminuria compared with age-matched control mice treated with the same agents at various time points (Figure 2f and g). Together, these findings indicate that Pod-*Vcl*-KO mice are more susceptible to glomerular injury.

### The loss of vinculin alters podocyte foot process morphology

**Figure 3:**
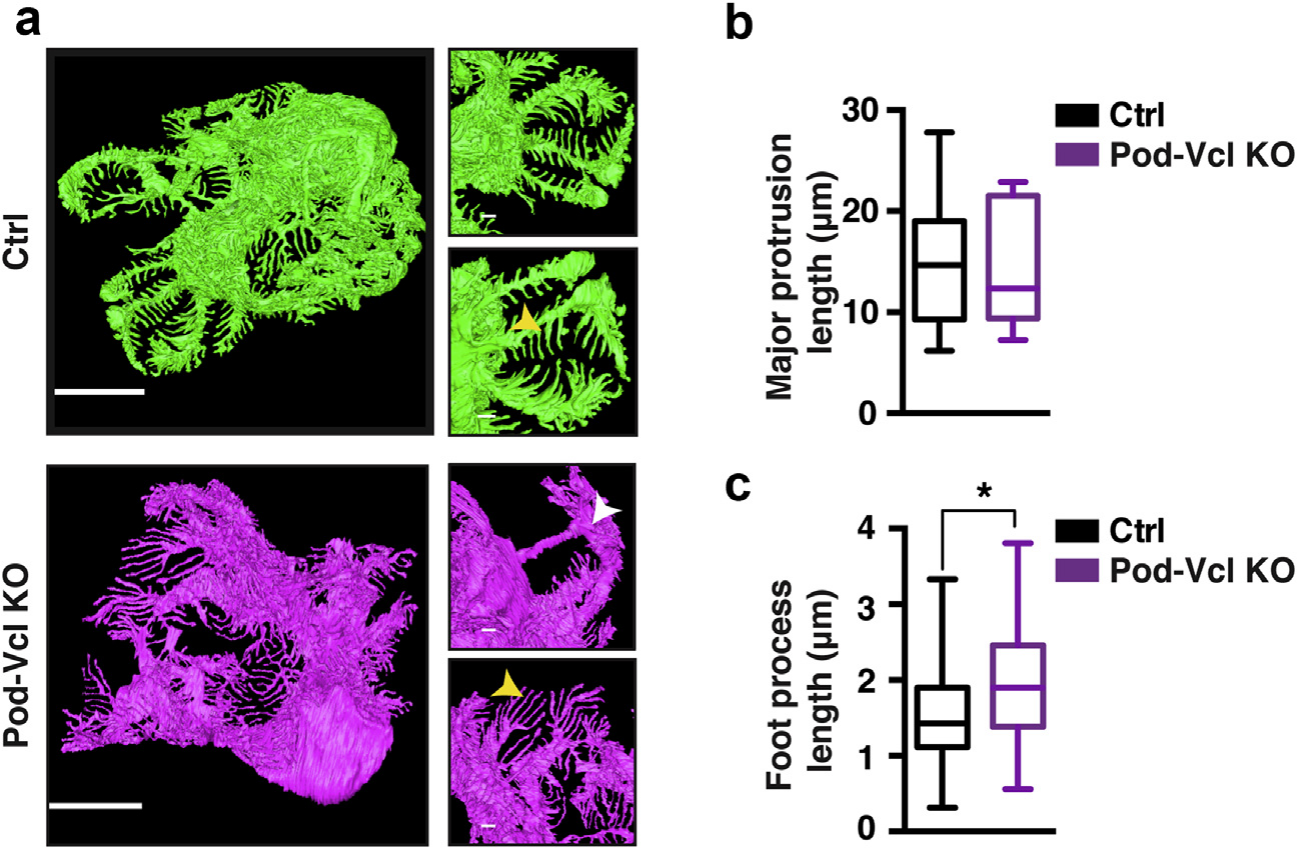
**Podocytes from Pod-*Vcl*-KO mice showed altered morphology of cellular protrusions *in vivo*.** 3View tracking of podocytes *in vivo* using serial block face scanning electron microscopy. White arrowhead highlights the rejoining of cellular protrusions. Yellow arrowheads highlight the foot process lengths. Bar = 10 um in the bigger images on the left, and 1 um in smaller images on the right (**a**). Length of major protrusions was measured within the modeled podocyte from the first branching point of the cell body to the final tip; n = 5 protrusions per cell; 3 podocytes from different animals were analyzed (**b**). Foot process length was measured from the last branching point to the end of the foot process (N = 60 processes per cell, N = 3 mice). *P < 0.001 based on a Student’s *t*-test (**c**). To optimize viewing of this image, please see the online version of this article at www.kidney-international.org.

**Figure S2:**
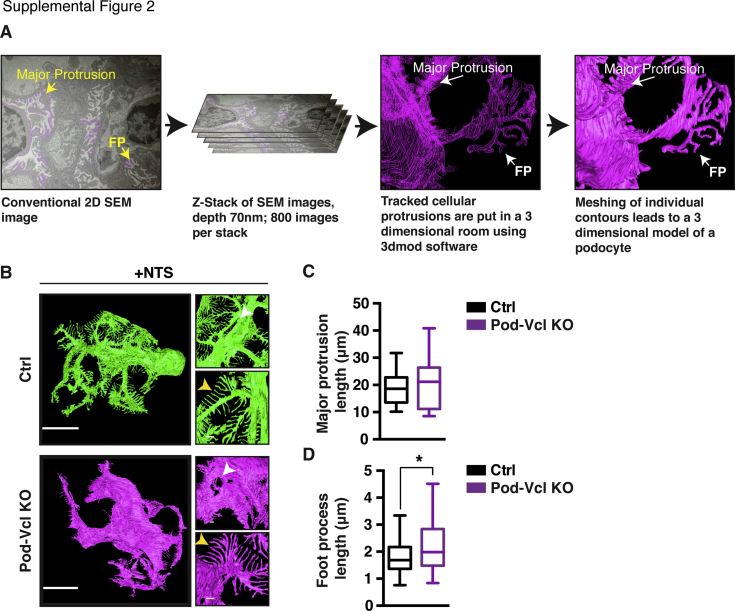
3View serial block-face scanning electron microscopy (SEM). Generation of a 3-dimensional model of podocytes using serial block-face SEM (**A**). 3D models of control (Ctrl) and Pod-*Vcl*-KO podocytes after challenge with nephrotoxic serum (NTS). White arrowhead highlights the branching defect, and the yellow arrowhead shows the difference in length of the foot processes. Note the branching defect in the ctrl podocytes after treatment with NTS (**B**). Quantification of major protrusion length (**C**). Quantification of foot process length; N = 3 cells modeled from different animals (**D**).

Podocytes form long, interdigitating foot processes *in vivo,* and these are indispensable for intact barrier function. To investigate whether vinculin plays a role in the regulation of podocyte morphology, we examined the number and length of podocyte foot processes in Pod-*Vcl*-KO mice *in vivo,* performing serial block-face scanning electron microscopy (SBFSEM). Feature tracking of intact podocytes revealed the complex morphology of these cells (Supplementary Figure S2A and Supplementary Movie S1). In contrast to control podocytes, we found that Pod-*Vcl*-KO podocytes had smaller cell bodies, with a branching phenotype in which processes merged soon after branching occurred (Figure 3a, white arrowhead; Supplementary Movies S2 and S3). Although the average length of the major protrusions was not different (Figure 3b), foot process length was significantly increased in Pod-*Vcl*-KO podocytes compared with control cells (Figure 3b, yellow arrowhead; quantified in Figure 3c). This increase in foot process length and the branching phenotype was exacerbated upon treatment with NTS (Supplementary Figure S2B–D). These data reveal that vinculin is required for maintenance of normal cellular protrusions and foot processes in the podocyte.

### Loss of vinculin increases FAK tyrosine phosphorylation in podocyte focal adhesions

**Figure 4:**
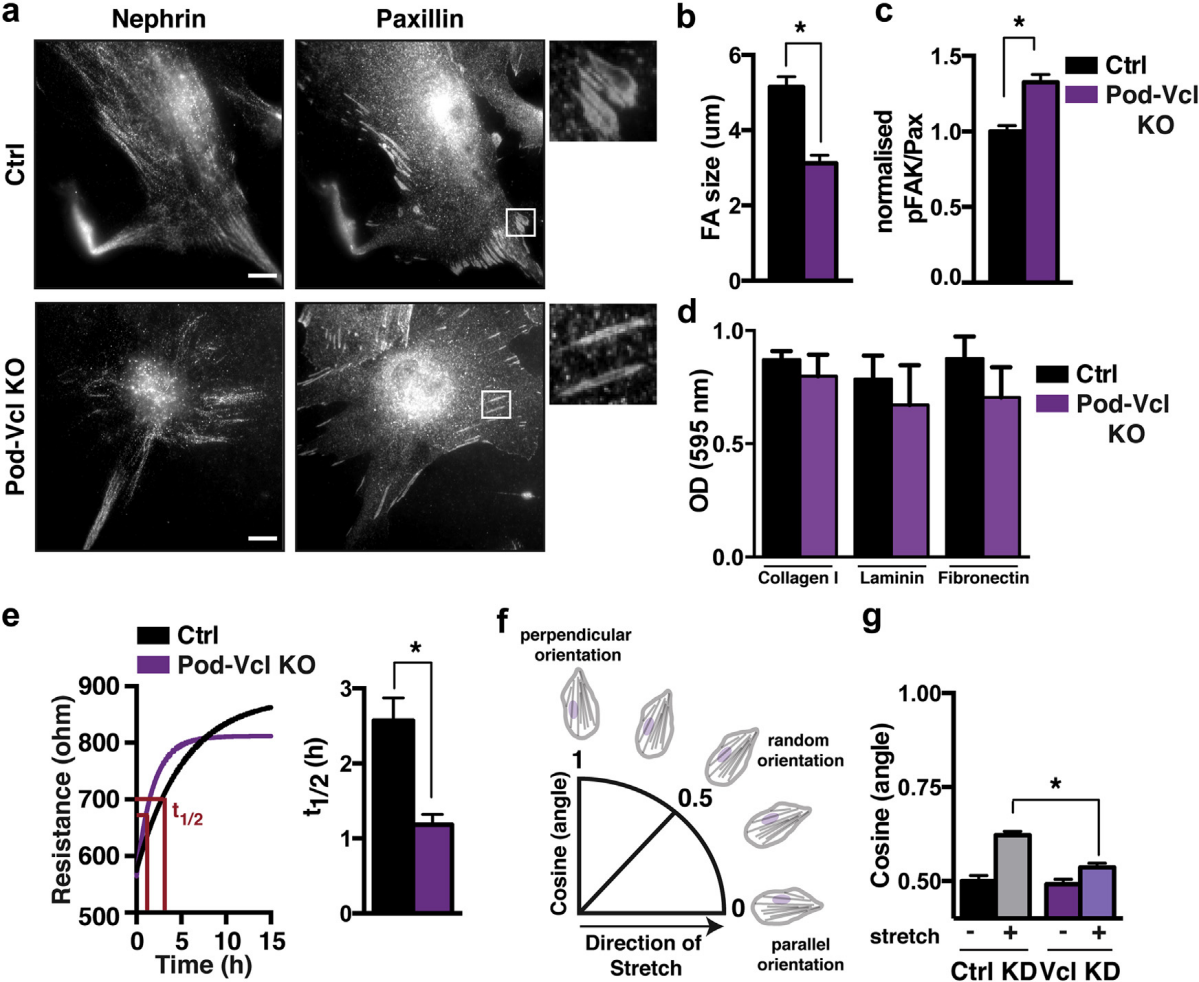
**Loss of vinculin impairs focal adhesion (FA) signaling.** Podocytes were isolated from control (Ctrl) and Pod-*Vcl*-KO mice and stained for paxillin. Nephrin staining was used to verify podocyte cell identity. Note that the FAs in Ctrl podocytes are bigger than those in vinculin-deficient podocytes (**a**). FA size was quantified by drawing around the adhesions and measuring the area in ImageJ. A total of 20–25 adhesions were analyzed, from 60 cells, in 3 independent experiments. *P < 0.001 (**b**). Phospho FAK (Y397) to paxillin levels were calculated performing ratio imaging. Focal adhesions were analyzed from 60 cells, in 3 independent experiments; *P < 0.01 (**c**). Adhesions assays, using crystal violet, were performed with Pod-*Vcl*-KO and Ctrl podocytes plated on collagen I, laminin, and fibronectin. Optical density (OD) was measured at 595 nm. N = 3 for each substrate (**d**). To investigate cellular migration, we performed a wounding assay using electrical cell substrate impedance sensing (ECIS), in which cells forming a cell monolayer insulate against an applied current. The left image shows a nonlinear regression of the increase in resistance after wounding. Half-time was calculated to compare migratory behavior of podocytes, filling the induced wound. The right graph shows quantified half-times. N = 18, pooled from 4 independent experiments (**e**). Cellular orientation of immortalized human podocytes was analyzed by calculating the cosine of the angle of cellular orientation regarding the direction of stretch. Cosine (angle) = 0.5 represents random cellular organization; cosine (angle) = 1 demonstrates perpendicular orientation toward the stretch direction, whereas cosine (angle) = 0 implies a parallel organization of the cells (**f**). Small interfering (si)RNA–mediated knockdown of vinculin and control knockdown using scrambled siRNA was induced in human immortalized podocytes, and the cosine of the angle was calculated. N = 300 from 3 independent experiments; *P < 0.05 (**g**). To optimize viewing of this image, please see the online version of this article at www.kidney-international.org.

**Figure S3:**
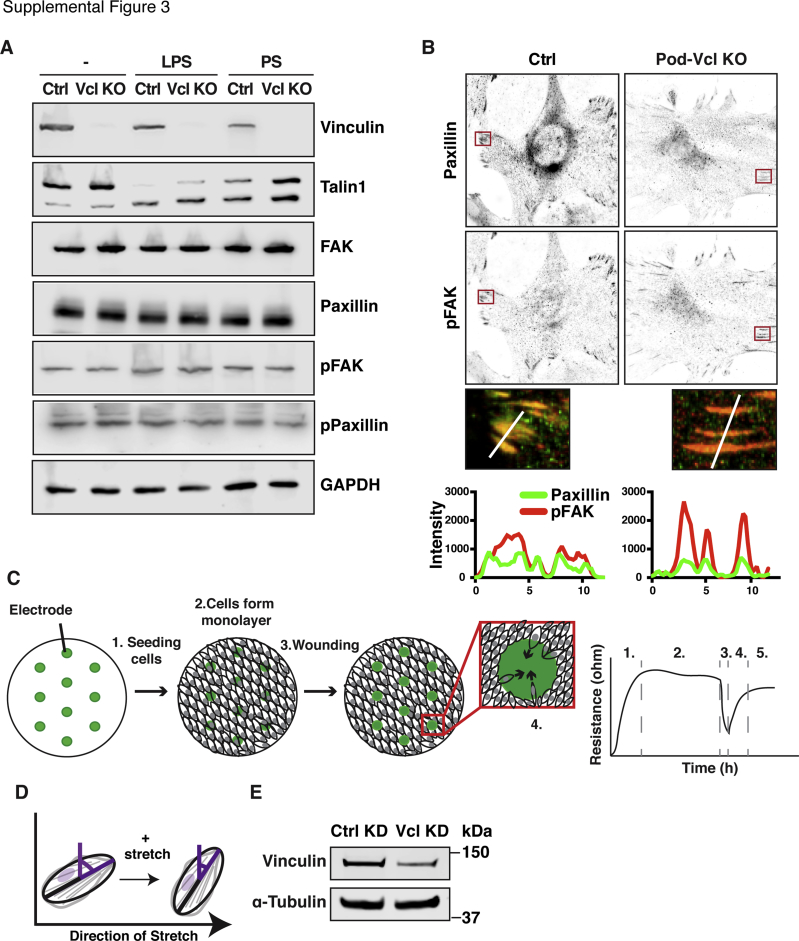
Vinculin deficiency increases phospho–focal adhesion kinase (pFAK) levels specifically at focal adhesions (FAs). Primary podocytes were isolated from control and Pod-*Vcl*-KO mice and treated with lipopolysaccharide (LPS) and protamine sulfate (PS). Western blot was performed to determine protein levels of FA proteins, vinculin, talin1, FAK, paxillin, pFAK, phospho-Paxillin (pPaxilin), and the housekeeping gene glyceraldehyde 3-phosphate dehydrogenase (GAPDH; **A**). Immunofluoresecence ratio imaging was performed to determine phopho-FAK levels relative to paxillin protein levels specifically at FAs. Line profile highlights elevated phospho-FAK levels to paxillin levels at FAs in podocytes isolated from Pod-*Vcl*-KO mice compared to control (Ctrl) podocytes (**B**). Schematic illustration of a wounding assay: A single electrical cell substrate impedance sensing (ECIS) well carries 10 electrodes, where resistance is measured. Following cell plating, resistance increases (1.) and finally plateaus (2.) when cells form a monolayer. Cells located on the electrode are wounded by application of a high current, leading to local cell death and a drop in resistance (3.). Increased migration and proliferation result in increasing resistance (4.). When a confluent cell monolayer is re-established, resistance plateaus (5.). The lower graph shows a representative curve progression of the ECIS resistance measurement, including a wounding assay (**C**). Schematic demonstration of the analysis of the stretching assay: After application of the stretching cycles, cells were fixed and stained with 4′,6-diamidino-2-phenylindole DAPI and phalloidin. An ellipse was constructed into the cells, and the angle was measured using ImagJ (**D**). Small interfering (si)RNA-induced knockdown of vinculin was verified using western blot analysis (**E**). α-tubulin was used as loading control. Upon stretch application, cells organize themselves so that they are perpendicular to the direction of stretch.

Vinculin is a known component of the consensus adhesome^28^ and plays a critical role in signal transduction from FAs^3^ to the actin cytoskeleton.^29^, ^30^ To investigate the role of vinculin in the regulation of podocyte FAs, we analyzed control and Pod-*Vcl*-KO podocytes, cultured on the GBM substrate collagen IV. Paxillin immunofluorescence revealed that the FAs of nephrin-positive control podocytes had an average area of 5 μm^2^, whereas those from Pod-*Vcl-*KO podocytes were significantly smaller, with an average size of 3 μm^2^ (Figure 4a; quantified in Figure 4b). Western blotting analysis revealed no differences in total cellular expression levels of FA proteins between control and Pod-*Vcl*-KO podocytes (Supplementary Figure S3A). Next, we examined whether the decreased FA size in Pod-*Vcl*-KO podocytes was associated with altered FA signaling, by comparing the ratio of phosphorylated FAK (pFAK) at tyrosine 397 to endogenous paxillin in Pod-*Vcl-*KO podocytes and control podocytes, using ratiometric imaging.^31^ These images revealed increased pFAK to paxillin levels in Pod-*Vcl-*KO podocytes, compared with ratios in control podocytes (Figure 4c; Supplementary Figure S3B). The total cellular protein levels of pFAK remained unchanged in Pod-*Vcl*-KO podocytes (Supplementary Figure S3A), highlighting the fact that vinculin-dependent FAK phosphorylation and signaling is site-specific.

Both vinculin and FAK have been shown to regulate the strength of adhesion of cells to ECM.^32^, ^33^ To investigate whether vinculin regulates adhesion strength in podocytes, we performed adhesion assays, plating Pod-*Vcl-KO* and control podocytes on collagen I, laminin, and fibronectin. Regardless of the substrate, no significant difference in adhesion was detected between control and Pod-*Vcl*-KO podocytes (Figure 4d). Signals from FAs control a diverse repertoire of cellular functions, such as migration and proliferation.^21^, ^34^ Vinculin, as well as FAK, has been shown to influence the motile behavior of cells.^32^, ^35^ Therefore, to investigate whether vinculin affects podocyte cell migration, we performed a wounding assay using electrical cell-substrate impedance sensing. In this assay, the transepithelial resistance of a cell monolayer against an applied current is measured. Inducing a wound to the cell monolayer by applying a high current locally induces cell death in a defined area. The induced wound led to a rapid drop in transepithelial resistance, which was followed by an increase in resistance as cells migrated and repopulated the gap. Filling the gap resulted in a resistance plateau when the epithelial barrier was fully re-established (Supplementary Figure S3C). Pod-*Vcl*-KO podocytes showed decreased half-times following the rise of resistance after wounding (Figure 4e), indicating faster migration compared with control podocytes.

### Loss of vinculin impairs podocyte mechanosensing

The kidney is a force-loaded organ, requiring appropriate mechanosensing to facilitate normal filtration. FAs are critical for sensing of external stimuli, and vinculin has been proposed to have a key role in this process. To investigate the extent to which vinculin is involved in sensing and adapting to varying forces in podocytes, we performed force-sensing assays whereby immortalized human podocytes, transfected with small interfering (si)RNA targeting vinculin, were seeded on flexible polydimethylsiloxane (PDMS) membranes and exposed to cyclic stretching for 25 minutes (10% strain at 1 Hz). Cellular responses were recorded, and the cellular re-orientation with respect to the direction of stretch was analyzed (Supplementary Figure S3D) by measuring the cosine of the measured angle (Figure 4f). Measurements revealed that podocytes transfected with scrambled siRNA (siCtrl) re-organized perpendicular to the stretch axis, as has been reported for other cell types^36^ (Figure 4g). Podocytes transfected with siRNA targeting vinculin (siVcl) (Supplementary Figure S3E) resulted in a significantly reduced ability to respond to stretching forces (Figure 4g). From these data, we conclude that vinculin plays an important role in the response of podocytes to stretching forces.

### ZO-1 localization at cell–cell junctions is impaired in *Pod-Vcl-KO* podocytes after injury

**Figure 5:**
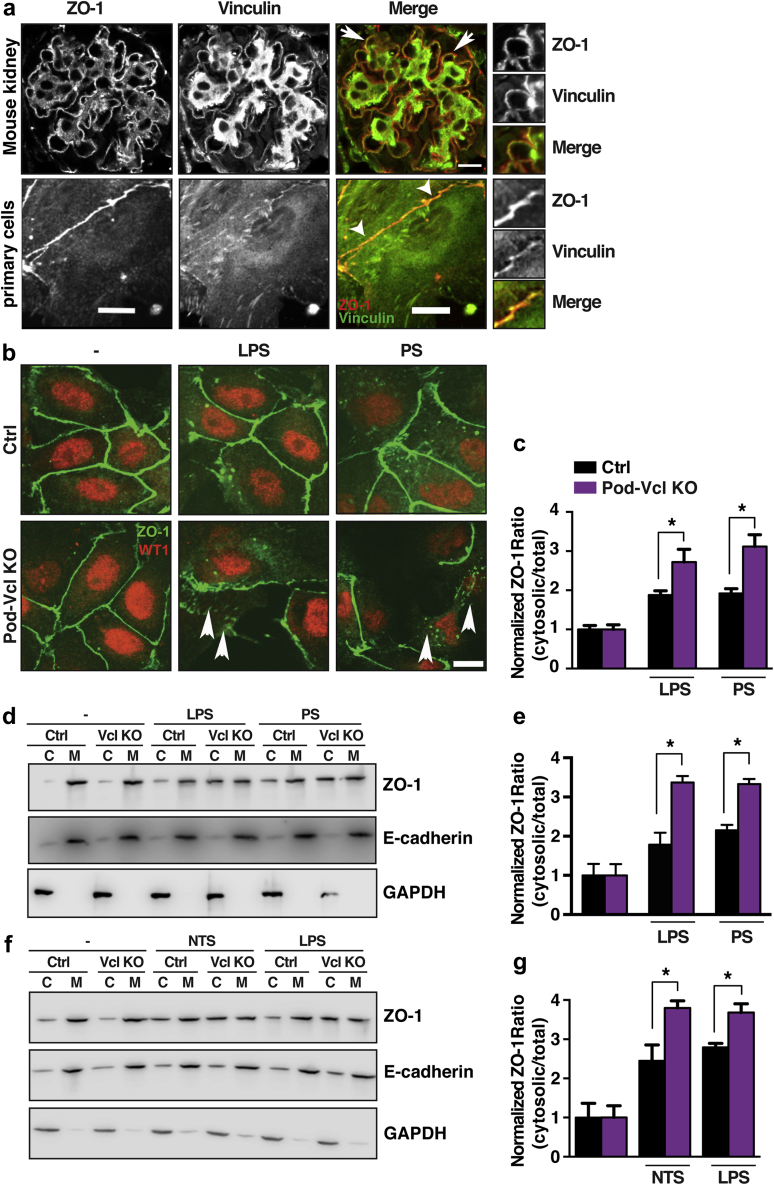
**Loss of *Vcl* in podocytes results in the redistribution of adherens junction protein zonula occludens (ZO)-1 to the cytosol.** Vinculin colocalizes with ZO-1 at cell–cell junctions (arrow) in wild-type mouse kidney tissue at age 8 weeks, and in primary podocytes (arrowheads) isolated from wild-type mice (**a**). Lipopolysaccharide (LPS) or protamine sulfate (PS) treatment in *Pod*-*Vcl-KO* podocytes results in an increase in cytosolic ZO-1, compared with control (Ctrl) podocytes. Arrowheads depict mislocalization of ZO-1 in Pod-*Vcl*-KO podocytes. Bar = 10 μm (**b**). Quantification of the distribution of ZO-1 by fluorescence intensity; n = 3; *P < 0.05; intensity was normalized to untreated conditions (**c**). Treatment with LPS or PS results in relocalization of ZO-1 to the cytosolic fraction in podocytes that lack vinculin (**d**). Quantification by densitometry of (**d**); n = 5; *P < 0.01 (**e**). Western blot of membrane and cytosolic fractions from podocytes isolated from Ctrl and Pod-*Vcl*-KO mice after treatment (*in vivo*) with LPS or NTS (**f**). Quantification by densitometry of (**f**); n = 5; *P < 0.01. Intensity was normalized to untreated conditions (**g**). C, cytoplasm; GAPDH, glyceraldehyde 3-phosphate dehydrogenase; M, membrane; NTS, nephrotoxic serum. To optimize viewing of this image, please see the online version of this article at www.kidney-international.org.

**Figure S4:**
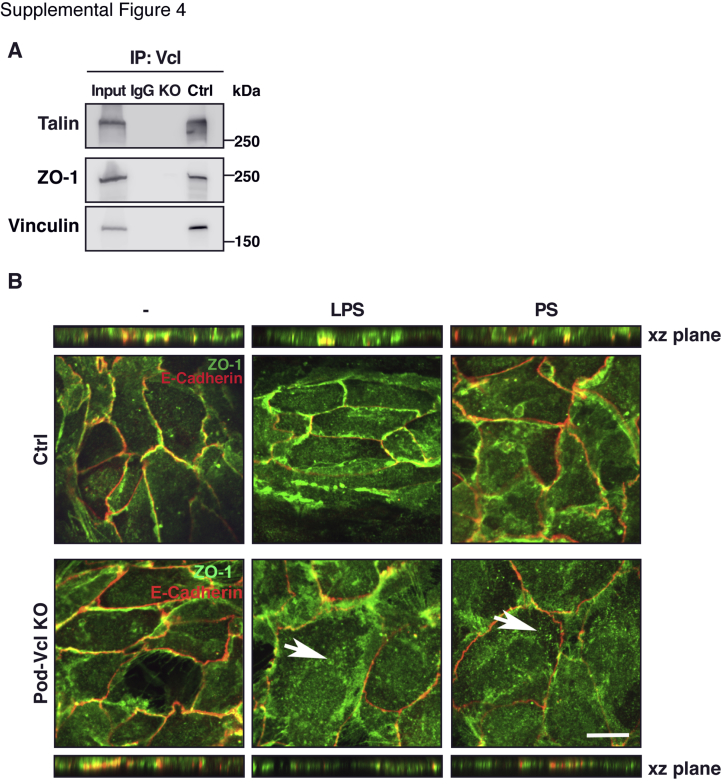
Vinculin controls zonula occludens (ZO)-1 localization upon injury. Co- immunoprecipitation of control and Pod-*Vcl*-KO podocytes shows vinculin association with ZO-1 in controls (ctrl), but not in IgG or vinculin KO podocytes (**A**). Representative XY and XZ images show that ZO-1- colocalization with membrane marker E-cadherin is reduced in Pod-*Vcl*-KO podocytes after lipopolysaccharide (LPS) and protamine sulfate (PS) stimulation (**B**).

As vinculin also plays an important role at sites other than FAs, we next examined how vinculin modulates intercellular adhesions. Given that ZO-1, a tight junction protein, has been shown to be required in podocyte health, and to interact with vinculin, we investigated whether vinculin helps to properly localize ZO-1. Using immunofluorescence, we found that vinculin co-localizes with ZO-1 both *in vivo* and *in vitro* (Figure 5a, white arrowhead). Vinculin co-localization was not found along the entire length of intercellular junctions but rather at discrete contact areas between two cells (Figure 5a, white arrowhead, lower panel). Additionally, vinculin immunoprecipitation revealed binding of ZO-1 to vinculin in control podocytes (Supplementary Figure S4A). To gain further insight into the potential role of vinculin in the regulation of these intercellular junctions, their integrity was determined by the localization of ZO-1 at cell–cell adhesions of Pod-*Vcl*-KO and control podocytes that were injured with either LPS or protamine sulfate (PS) stimulation. Quantitative immunofluorescence staining of primary podocytes stimulated with LPS or PS revealed an increased abundance of ZO-1 localization in the cytoplasm of Pod-*Vcl*-KO podocytes, in comparison with control podocytes (Figure 5b; quantified in Figure 5c; Supplementary Figure S4B.) Additionally, cell fractionation revealed significantly pronounced ZO-1 redistribution from the membrane to the cytoplasm after LPS- or PS-induced injury in Pod-*Vcl*-KO podocytes, compared with control podocytes (Figure 5d; quantified in Figure 5e). Further validating our findings, *in vivo*, podocytes isolated from Pod-*Vcl*-KO and control mice that were injected with either LPS or NTS revealed a significantly increased presence of ZO-1 in the cytoplasm of Pod-*Vcl*-KO, compared with control podocytes (Figure 5f; quantified in Figure 5g).

**Figure 6:**
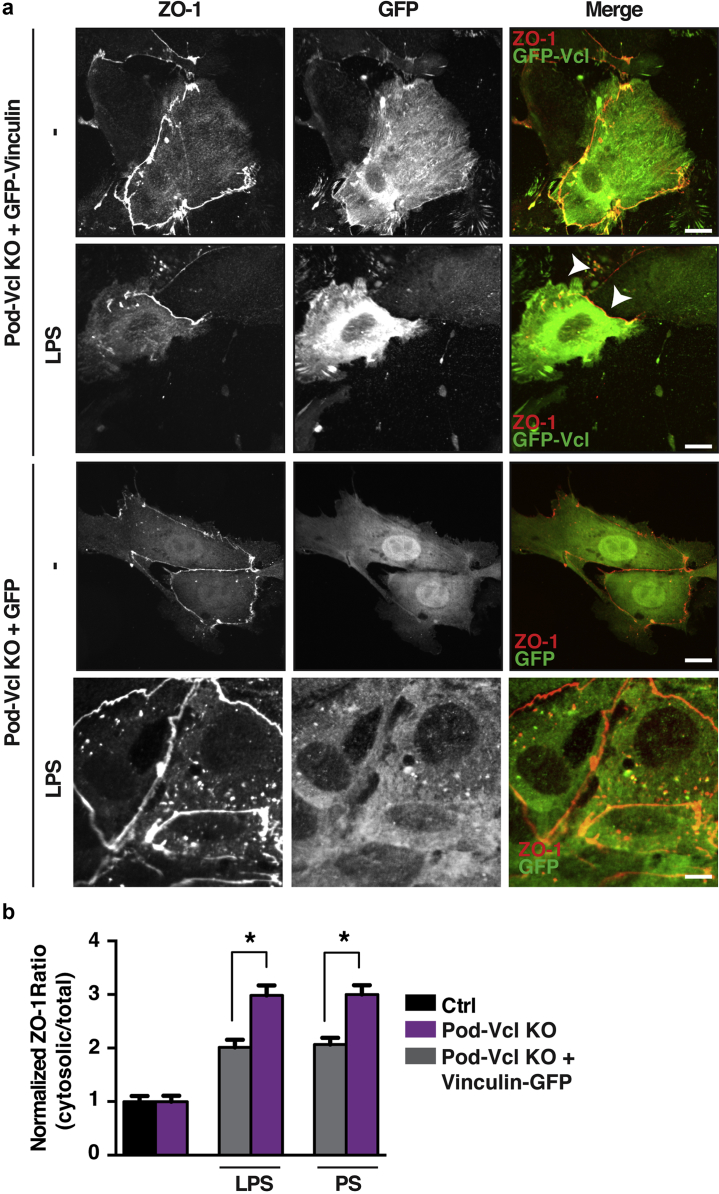
**Re-expression of green fluorescent protein (GFP)–*Vcl* in Pod-*Vcl*-KO podocytes rescues zonula occludens (ZO)-1 mislocalization after either lipopolysaccharide (LPS) or protamine sulfate (PS) treatment.** Representative image of Pod-*Vcl*-KO primary podocytes re-expressing GFP-vinculin shows that zonula occludens (ZO)-1 is at the cell–cell junctions, and this persists after the LPS challenge (white arrowheads). Note the ZO-1 mislocalization in adjacent, untransfected Pod-*Vcl*-KO podocytes serving as an internal control (arrowhead). Bar = 10 μm. GFP alone transfection was also performed as a control (ctrl). Note that the bottom panel stimulated with LPS is at x100 (**a**). Quantitative analysis of the redistribution of ZO-1 is represented by comparing the cytoplasmic to the total cellular immunofluorescence intensity after either LPS or PS treatment in mouse GFP–vinculin expressing and nonexpressing primary podocytes; n = 3; *P < 0.05 (**b**). To optimize viewing of this image, please see the online version of this article at www.kidney-international.org.

To further confirm the role of vinculin in stabilizing intercellular adhesions in podocytes, we performed rescue experiments and re-expressed vinculin into Pod-*Vcl*-KO podocytes. Our results reveal that expression of green fluorescent protein–vinculin in Pod-*Vcl*-KO podocytes partially restored ZO-1 localization at cell–cell junctions even after podocyte injury. Quantification showed a 25% reduction of ZO-1 intensity in the cytoplasm of rescued cells in comparison with untransfected Pod-*Vcl*-KO podocytes treated with LPS or PS (Figure 6a; quantified in Figure 6b). Taken together, these experiments reveal that vinculin is pivotal in stabilizing cell–cell adhesions in podocytes after glomerular injury.

### Vinculin levels are altered in human glomerular disease

**Figure 7:**
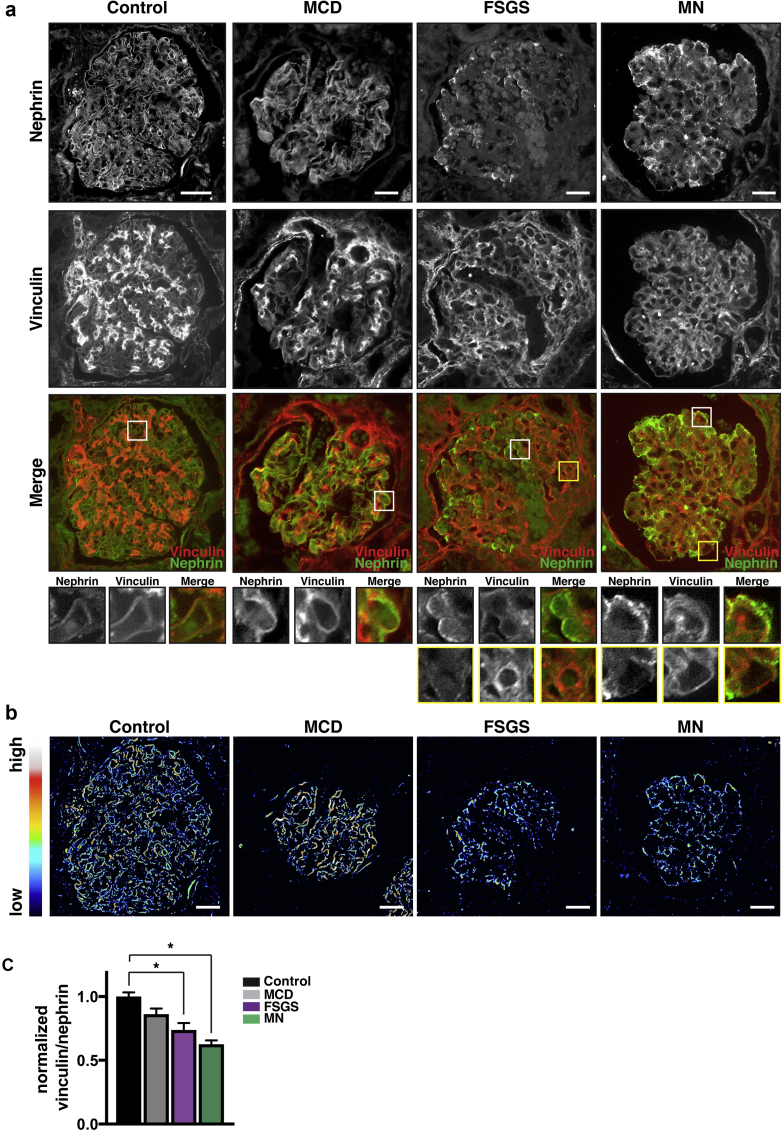
**Vinculin is reduced in human glomerular disease.** Panels show localization of nephrin and vinculin in human kidney biopsy sections from patients with focal segmental glomerulosclerosis (FSGS), minimal change disease (MCD), or membranous nephropathy (MN). The altered localization of vinculin and nephrin is shown in zoomed-in images at the bottom of the panel. In FSGS and MN in particular, there appeared to be less vinculin in capillary loops with positive nephrin staining, compared with regions where nephrin localization was reduced, and here vinculin was increased (yellow boxes). Bar = 20 μm (**a**). Ratiometric imaging was performed by selecting the nephrin signal as a mask and then relating vinculin to nephrin intensity. Bar = 30 μm (**b**). Quantification of (**b**): All histological subtypes were analyzed from n = 3 biopsy samples. *P < 0.001 (**c**). To optimize viewing of this image, please see the online version of this article at www.kidney-international.org.

**Figure S5:**
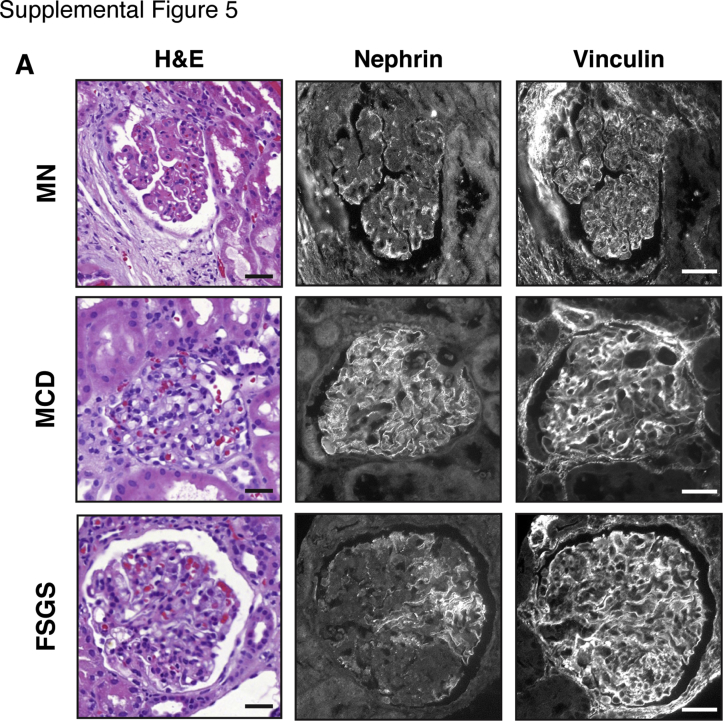
Membranous nephropathy (MN) and focal segmental glomerulosclerosis (FSGS) sections show altered hematoxylin and eosin (H&E) and nephrin staining. Serial sections from patients with MN, minimal change disease (MCD), and FSGS were stained for H&E and in parallel for nephrin and vinculin. Bar = 20 μm (**A**).

To investigate the importance of vinculin in human disease processes, we analyzed kidney biopsy sections from patients who had FSGS, MCD, or MN, and compared them to healthy control tissue. The localization of nephrin was similar in control and MCD sections and showed an even distribution across glomeruli (Figure 7a). In contrast, the localization of nephrin in MN and FSGS samples was more focal and punctate, and regions of reduced nephrin expression had increased ECM deposition (Supplementary Figure S5). Vinculin localized to nephrin-positive capillary loops in all analyzed disease cases (Figure 7a, white boxes). However, in FSGS, we detected reduced vinculin localization to nephrin-positive capillary loops, compared with capillary loops, with reduced nephrin staining (Figure 7a—compare white and yellow boxes). To analyze overall vinculin localization to capillary loops in glomerular diseases, we performed ratiometric imaging, quantifying vinculin levels relative to nephrin levels specifically in nephrin-positive areas (Figure 7b). The blue-colored nephrin-positive masks, in both FSGS and MN sections, indicate a low vinculin-to-nephrin ratio, whereas in control and MCD sections, we detected red/white color patterns, indicating a higher vinculin-to-nephrin ratio in these glomeruli (Figure 7b; quantified in Figure 7c). Taken together, these data demonstrate that with human glomerular disease, marked differences are present in the level and localization of vinculin.

## Discussion

Vinculin is known as a mechanosensitive regulator of cell–matrix adhesions and cell–cell junctions. In this study, we found that vinculin has a role in maintaining podocyte barrier integrity both *in vivo* and *in vitro*. We report the following: (i) Pod-*Vcl*-KO mice develop proteinuria upon challenge; (ii) defects in podocyte cell protrusion/foot process morphology occur in Pod-*Vcl*-KO mice; (iii) altered adhesion signaling with impaired FAK phosphorylation occurs in Pod-*Vcl*-KO podocytes; (iv) disrupted intercellular junctions with mislocalization of the key junctional protein ZO-1 are detected in Pod-*Vcl-*KO podocytes; and (v) analysis of human kidney sections revealed that vinculin localization is altered in glomeruli of FSGS, MCD, and MN samples, compared with control samples. Overall, our findings highlight the importance of vinculin in the regulation of the glomerular filtration barrier in response to injury in both mouse and human kidneys.

Vinculin is a core component of the FA protein complex, where it is exposed to tension.^15^ In this study, we show that vinculin is a critical regulator of tension-sensing in podocytes; vinculin-deficient podocytes showed a significant decrease in ability to respond to cyclic stretching. These data are in keeping with studies in fibroblasts showing that vinculin (and its link to the actin cytoskeleton) is important for not only sensing of stretching forces^30^ but also transduction of forces in FAs.^20^, ^37^, ^38^ In addition to mechanotransduction, vinculin controls adhesion signaling by coordinating adhesion signaling proteins.^30^ This ability largely depends on its intimate relationship with talin,^39^, ^40^ which also is required to maintain glomerular barrier integrity; podocyte-specific KO of talin 1 causes severe glomerular dysfunction and early kidney failure.^7^ FAK is also a core constituent of FAs, binding to talin^41^ and regulating FA turnover.^42^, ^43^ In this study, we found that FAK phosphorylation (Y397) is increased in FAs of *Pod-Vcl-KO* podocytes (Figure 4c). This increase in FAK phosphorylation may explain the increase we observed in cell motility in Pod-*Vcl*-KO podocytes, because increased FAK phosphorylation (in conjunction with Src and paxillin phosphorylation) has been associated with an increase in protrusive activity^44^ through increased Rac1 activity.^45^, ^46^

We observed significant differences in the organization of cellular protrusions between *Pod-Vcl- KO* and control podocytes *in vivo,* in which we observed an increased number of foot processes in Pod-*Vcl*-KO podocytes. This highlights the role of vinculin in the organization of the actin-dependent cellular extensions. Furthermore, LPS/PS-induced injuries *in vivo* were associated with more proteinuria in the Pod-*Vcl*-KO, compared with control mice expressing vinculin, suggesting its importance in maintaining the integrity of the barrier function.

Vinculin not only controls FA stability, but also regulates cell–cell adhesions.^12^ In epithelial cells, vinculin localization to cell–cell junctions is driven in a force-dependent manner.^47^ Considering the consistently high transcapillary pressure that podocytes endure as they encounter 180L of plasma daily, we suggest that vinculin functions in a force-dependent way in podocytes, where its recruitment helps to maintain the integrity of the glomerular filtration barrier. In this regard, the mild phenotype observed in the Pod-*Vcl*-KO mice was initially surprising. This finding suggests that vinculin is dispensable during development of the kidney but plays a more important role at later stages, or when the kidney is challenged and needs to recover after (or be protected from) injury. This possibility is in line with our observations showing that defects in barrier function become more evident upon challenge with LPS and NTS (Figure 2e and f). This hypothesis is also consistent with reports showing that cells are able to form cell–matrix adhesions and cell–cell junctions in the absence of vinculin and that deficiencies in cellular responses become evident only upon challenge (e.g., application of forces).^30^, ^47^, ^48^ In addition, we found that 38% of aged mice had large glomerular cysts. Cystic kidney disease is associated with a growing number of mutations in the components of primary cilia. Glomerulocystic disease overlaps with this disease spectrum, and the absence of vinculin may be relevant for normal mechanosensing of flow in the glomerulus in aged mice.

Intercellular junctions are critical for the maintenance of glomerular filtration,^49^ and we detected decreased glomerular barrier function upon glomerular injury in Pod-*Vcl*-KO mice (Figure 2), suggesting that vinculin contributes to these junctions in podocytes. The junctional protein ZO-1 appears to have a particular role in the cell–cell junctional mediated barrier function, as our data show that in Pod-*Vcl-*KO podocytes, ZO-1, upon induction of injury, fails to localize to intercellular junctions, a phenotype that can be rescued by the re-expression of vinculin in these cells (Figure 6). The regulatory effect in cell–cell adhesions would be in line with other reports, in which vinculin was found to have a role in the communication between cells by binding to ZO-1, thus stabilizing gap junction formation.^25^ Podocyte-specific loss of ZO-1 in mice results in severe proteinuria, foot process effacement, and glomerulosclerosis.^26^ Hence, the altered localization of ZO-1 in our experiments could be a factor in the reduction of barrier function after LPS and NTS challenge (Figure 5). The significance of vinculin localization to specific areas along a cell–cell contact in podocytes is yet to be determined, but it could represent a contribution to an “adhesion zipper,” previously described as an initial step in adherens junction formation of keratinocytes.^50^ Additional experiments are needed to test this model and further dissect the mechanism by which vinculin contributes to glomerular barrier function.

In conclusion, we report that vinculin has a key role in the regulation of podocyte-mediated glomerular filtration. Our data suggest that vinculin has a protective function and is important for maintenance of the barrier function after injury. This model is in line with our data from human biopsies demonstrating that vinculin localization is altered in glomerular disease. With the advent of whole-exome and whole-genome sequencing across an ever-increasing number of clinical cohorts with kidney disease, genetic variants in vinculin may shed further light on the role of vinculin in human disease.

## Concise Methods

### Mice

*Podocin-Cre* recombinase mice were obtained from the Jackson Laboratory, Bar Harbor, Maine (B6.Cg-Tg(NPHS2-Cre)295Lbh/J, stock number 008205). The mice carrying the floxed vinculin construct were obtained from Robert Ross, University of California, San Diego.^24^ Analyzed Pod-*Vcl*-KO and control littermate mice were on a C57/Bl6 background, which is relatively resistant to glomerular disease. Urine samples were collected from the Pod-*Vcl*-KO and littermate *Vcl* ^*fl/fl*^ controls at indicated time.

### NTS and LPS treatment

Treatment of mice with rabbit anti-mouse GBM antibody (NTS), generated by Lampire Biological Laboratories, was performed as previously described in our laboratory.^7^, ^51^ Briefly, mice were preimmunized 6 days prior to administration of NTS, via i.p. injection of 250 μg of rabbit IgG (Jackson Immunoresearch Laboratories, Bar Harbor, ME) in 250 μl of 1:1 emulsion with complete Freund's adjuvant (Sigma-Aldrich, St Louis, MO). Glomerulonephritis was induced with 200 μg of NTS using retro-orbital injection as previously described.^7^, ^51^ Preimmune rabbit serum was used as a negative control. For the LPS-induced animal model, mice were given an i.p. injection either with or without LPS (10 μg/g body weight, 1mg/ml in sterile phosphate buffered saline). Animal perfusions with 4% paraformaldehyde (PFA) were carried out as previously described.^51^ Pod-*Vcl*-KO mice and control mice (age 6 to 8 weeks) were used for these experiments. Urinary albumin excretion was measured at various time points.

### Electron microscopy

As previously described,^52^ we used transmission electron microscopy (TEM) and serial block-face scanning EM (SBF-SEM) to investigate glomerular ultrastructure. Tissue samples were fixed for at least 1 hour in a mix of 2% formaldehyde and 2.5% glutaraldehyde in 0.1 M sodium cacodylate buffer (pH 7.4). The samples were post-fixed with reduced osmium (1% OsO_4_ and 1.5% K_4_Fe(CN)_6_) for 1 hour, then with 1% tannic acid in 0.1 M sodium cacodylate buffer for 1 hour, and finally with 1% uranyl acetate in water overnight. The specimens were dehydrated with alcohols, infiltrated with resin (TAAB LV, Taab Laboratories, Reading, United Kingdom), and polymerized for 12 hours at 60 ° C. Ultrathin 70-nm sections were cut with a Leica Ultracut S ultramicrotome (Leica, Wetzlar, Germany) and placed on formvar/carbon-coated slot grids. The grids were observed in a Tecnai 12 Biotwin transmission electron microscope (ThermoFisher Scientific, Waltham, MA) at 80 kV. EM data were screened for matrix characteristics using Fiji/ImageJ software (version 1.46r; National Institutes of Health, Bethesda, MD). GBM thickness was quantified in five regions per observation and reported as mean values. For SBF-SEM, the polymerized samples were trimmed, glued to aluminum pins, and sputter coated with gold/palladium (60 seconds at standard settings). Automated sectioning (60 nm) was completed in a Gatan 3View (Gatan Inc, Pleasanton, CA). The images were taken with FEI Quanta 250 FEG SEM (ThermoFisher Scientific) at 3.8kV accelerating voltage and 0.4 Torr chamber pressure, with a pixel size of 12 nm. Models were generated from SBF-SEM using IMOD 3Dmod software (http://bio3d.colorado.edu/imod/download.html.), developed by David Mastronade and coworkers at the Boulder (CO) Laboratory for 3-D Electron Microscopy.

### Study approval

All animal experiments were approved by the relevant institutional regulatory board as follows: the University Committee on the Use and Care of Animals Institutional Review Board approved work at Yale University, and all work was conducted in accordance with the principles and procedures outlined in the National Institutes of Health Guide for the Care and Use of Laboratory Animals (8th edition, 2011). In Manchester, the project license 40/3409 was reviewed and approved by the University of Manchester Animal Welfare and Ethical Review Board, and all procedures were carried out in accordance with the European Directive 2010/63/EU governed by the Home office in the United Kingdom. Ethical approval was obtained to use archival pediatric biopsy samples (REC reference: 14/WA/0083), normal human tissue, and archival adult biopsy samples (REC reference: 16/NW/0119).

### Statistical analysis

All measurements are shown as mean ± SEM. Box plots indicate maximum and minimum data points (whiskers). Bar chart error bars represent SEM. Statistical analysis was performed using 2-tailed Student’s *t*-test or 1-way analysis of variance (SigmaStat, version 3.1.1). *P* < 0.05 was considered statistically significant.

Antibodies and reagents and all other methodology are described in the Supplemental Experimental Procedures.

## Disclosure

All the authors declared no competing interests.

## Supplementary Material

Supplemental Experimental Procedures Antibodies and reagents, urinary albumin and creatinine quantification, isolation of murine glomeruli and cell culture, knockdown experiments, Electric Cell-substrate Impedance Sensing (ECIS), measurement of albumin flux, stretching assay, analysis of membrane to cytosol transfer of ZO-1, Western blotting, PCR, quantification of the number of foot processes from electron micrographs, immunofluorescence, ratiometric imaging analysis, and immunoprecipitation.

Movie S1 Modeling of podocytes *in vivo.* Three adjacent podocytes from a control mouse were modeled from 800 sections captured by serial block-face scanning electron microscopy. The movie illustrates the complex cellular architecture of podocytes and their interdigitating foot processes.

Movie S2 Control podocyte. Serial block-face scanning electron microscopy and modeling were used to generate this 3D model of a control podocyte. It demonstrates the branch hierarchy of the cell body, from major protrusions to fine foot processes.

Movie S3 Podocyte-*Vcl*-knockout (Pod-*Vcl-*KO) podocyte. Serial block-face scanning electron microscopy and modeling were used to generate this 3D model of a Pod-*Vcl-*KO podocyte. Defects were detected in the branching of major protrusions, and foot processes appeared longer in the KO podocytes than in control cells.
