# Supplementary material for: Vinculin is required to maintain glomerular barrier integrity
Source: Kidney Int. 2018 Mar;93(3):643–55. doi: 10.1016/j.kint.2017.09.021 (PMC5846847; doi:10.1016/j.kint.2017.09.021)
Supplement: Supplemental Experimental Procedures — Antibodies and reagents, urinary albumin and creatinine quantification, isolation of murine glomeruli and cell culture, knockdown experiments, Electric Cell-substrate Impedance Sensing (ECIS), measurement of albumin flux, stretching assay, analysis of membrane to cytosol transfer of ZO-1, Western blotting, PCR, quantification of the number of foot processes from electron micrographs, immunofluorescence, ratiometric imaging analysis, and immunoprecipitation. [file mmc1.docx]

**Supplemental Experimental Procedures**

**Antibodies and reagents**

Monoclonal antibodies used were against actin (clone AC-40; Sigma-Aldrich, Poole, UK) mtHSP70 (MA3-028; Fisher scientific, Waltham, USA), vinculin (hvin1; Sigma, St.Louis, USA), ZO-1 (a gift kindly provided by Dr. Sachiko Tsukita at Osaka University, Osaka, Japan) ^1^, FAK antibody clone 77/FAK (BD Biosciences, San Jose, USA), pFAK antibody clone 349 (Thermo Fisher, Waltham, MA, USA), paxillin antibody clone 165 and 349 (BD Biosciences, San Jose, USA)), talin1 clone 97H6 (ABD Serotec) and 8D4 (abcam, Cambridge, UK), E-Cadherin antibody clone 36/E-Cadherin (BD Biosciences), GAPDH antibody clone 14C10 (Cell signaling, Cambridge, UK), γ-Tubulin antibody (Sigma), GFP antibody clone 7.1 and 13.1 antibody (Roche Life Science). Polyclonal antibodies were used against nephrin (a gift kindly provided by Dr. Yutaka Harita at University of Tokyo, Tokyo, Japan) ^2^ and from Acris (Herford, Germany). Secondary antibodies conjugated to IRDye 680 (Rockland Immunochemicals, Glibertsville, PA, USA) were used for western blotting. Alexa Fluor 488 goat anti-rabbit IgG antibody, Alexa Fluor 594 goat anti-rabbit IgG antibody, Alexa Fluor 488 goat anti-mouse IgG antibody, Alexa Fluor 594 goat anti-mouse IgG antibody were purchased from Invitrogen (California, USA). pGFP(C3)-*Vcl* plasmid was obtained from Addgene (Cambridge, USA). Cell culture media was purchased from Invitrogen. Collagen type I, IV, laminin, and fibronectin were purchased from BD Biosciences (San Jose, USA). Protamine sulfate and lipopolysaccharide (LPS) (Escherichia coli 0111:B4) were purchased from Sigma-Aldrich. ChromPure rabbit IgG and CFA were purchased from Jackson Immunoresearch Laboratories and Sigma-Aldrich, respectively. Texas red-labeled BSA was purchased from Molecular Probes (Eugene, USA).

**Urinary Albumin and Creatinine quantification**

Urine albumin levels were measured qualitatively and in duplicate using an albumin ELISA quantitation kit according to the manufacturer’s protocol (Bethyl Laboratories Inc.), and the absorbance read at 450 nm (glomax multi detection system; Promega) as previously described ^3^. Urine creatinine was measured in duplicate for each sample with an ELISA quantitation kit (Bioassay Systems) at an absorbance of 490 nm (Bio-Rad Microplate Reader). The urine albumin concentration of each sample was normalized to its urine creatinine concentration, expressed as the ratio of albumin (μg) / creatinine (mg).

**Isolation of murine glomeruli and cell culture**

Isolation of glomeruli was performed as described before.^3, 4^ The glomeruli were harvested by Percoll gradient and dynabead perfusion as previously described.^3^ In brief, kidneys were isolated, decapsulated and the medulla was removed. After cutting the kidney cortex into small pieces, the tissue was pressed through a 100µm cell strainer (Becton Dickinson, Oxford, UK) and washed with sterilized PBS. This crude isolate was then passed through a 70 μm cell strainer (Becton Dickinson, Oxford, UK), where glomeruli were caught. Glomeruli were washed with PBS and seeded onto 145 cm^2^ dishes. After 10 days of culturing, podocytes outgrowths were detected. Purity of the podocytes were >90% examining DAPI and WT1 co-staining.

Cells were cultured in RPMI 1640 medium with 10% FBS, 100U/ml penicillin, 100 µg/ml streptomycin, 100mM HEPES, 1mM sodium bicarbonate, 1mM sodium pyruvate.

A human immortalized human podocyte cell line (kind gift from Moin A Saleem) was used for knockdown experiments.^5^

**Knockdown experiments**

Scrambled siRNA and Vcl siRNA (s14762) were purchased from Thermo Fisher Scientific. Cells were transfected using Lipofectamine 2000 according to manufacturers instructions (Thermo Fisher Scientific). 50 picomol of siRNA were used and 2 rounds of knockdown were performed with one day of recovery, to guarantee protein knockdown for the duration of the ECIS experiments.

**Electric Cell-substrate Impedance Sensing (ECIS)**

The electrodes were prepared according to the manufacturer’s instructions (Applied BioPhysics) and coated with collagen IV (5 μg/ml). 150000 cells were seeded on 8W10E PET electrode arrays (ECIS cultureware, ibidi). Cells were held at 37°C with 5% CO_2_ and the media was exchanged at least every 24h. Resistance, Capacitance and Impedance were monitored at different frequencies. Overall barrier function was analysed at a frequency of 4000 Hz that in a monolayer is a measurement for the tightness of intercellular junctions. Cell spreading and the continuous growth towards the formation of a cell monolayer analyzed using the capacitance at a high frequency of 64000 Hz^6^.

**Measurement of albumin flux**

The transepithelial permeability of primary podcocytes isolated from control and *Vcl* specific knockout mice was assessed by measuring the passage of Texas Red-labeled BSA across the cell monolayer. The experiment was performed as described previously^7, 8^. Briefly, primary podocytes were cultured in transwell plates (0.4µm pore size, Corning Incorporated). Three days after cells achieved confluence, they were washed with PBS and the medium was replaced with serum-free RPMI medium in the apical compartment and the basolateral compartment. Texas Red-labeled BSA was loaded into the apical compartment at a final concentration of 100µg/ml. At the end of the incubation, fluorescence in the basaolateral compartment was collected and measured using a fluorescence reader (excitation =590 nm; emission =625 nm) at indicated experimental time intervals (Glomax multi detection system; Promega). A standard curve was plotted to calculate the amount of BSA. Five independent experiments were performed.

**Stretching assay**

Polydimethylsiloxane (PDMS) membranes were prepared, mixing Sylgard 184 silicone elastomer (Dow Corning, Michigan, USA) and Sylgard 184 elastomer curing agent in a 10:1 ratio. The mixture was stirred for 5 minutes and degased one hour. SThe silicone mixture was poured into a customized mold. The mold was placed in an oven at 65°C for 2.5h to cure the membranes. PDMS membranes were plasma cleaned (PDC-001, Harrick Plasma, Ithaca, NY, USA) according to manufacturer’s instructions and then coated with collagen IV (5μg/ml) and cells were seeded on the membranes 12 hours before stretch application. Stretching of the PDMS membrane was performed using a customized stretching instrument. 10 % strain at 1Hz was applied for 25 minutes in a unidirectional fashion. After stretching, cells were fixed and stained. Nephrin staining was used to identify podocytes. Cellular orientation was analysed with regards to the stretching direction. In brief, an ellipse was drawn through the cell body and the angle was measured using ImageJ 2.0.

**Analysis of membrane to cytosol transfer of ZO-1**

The experiment was performed as described previously^9, 10^. All procedures were performed at 4°C. Freshly isolated primary podocytes obtained from *Pod-Vcl-KO* mice and control littermates were cultured on collagen type-I coated dishes. After overnight serum starvation in RPMI medium, the podocytes were treated with LPS (10 µg/ml) for 12 hours or PS (300 µg/ml) for 1 hour, then harvested and homogenized in buffer (10 mM Tris-HCl, pH 7.5; 1 mM EGTA; 1mM MgCl_2_) containing protease inhibitors (Roche Diagnostics, IN, USA). Cells were disrupted by passing through a 22-gauge needle, and the lysates were centrifuged at 4300xg at 4°C for 10mins. The supernatant was centrifuged at 100,000xg at 4°C for 1 hour using a Beckman Optima TLX ultracentrifuge. The supernatant was collected, and the membrane fraction pellet was washed with hypotonic buffer (10mM Tris, pH 7.5; 1.5mM MgCl_2_;10mM KCl; 0.5mM DTT) three times, then dissolved with buffer (50 mM Tris-HCl, pH 7.5; 140 mM NaCl; 10% Glycerol; 1% Triton X-100) with 1% SDS and protease inhibitors. Both fractions were reconstituted in SDS-PAGE sample buffer and analysed by western blot. Protein band intensities were analysed using NIH imageJ software. Five different experiments were performed. For the glomerular samples, fresh glomeruli from *Pod-Vcl-KO* mice and control littermates following LPS injection or NTS injection were isolated using the Percoll (GE Heslthcare Bio-Sciences AB, Sweden) gradient method as previously described^3^ and underwent membrane fractionation as above. Five mice samples each group were performed.

**Western blotting**

Following SDS-PAGE, resolved proteins were transferred to nitrocellulose membrane (Whatman, Maidstone, UK) as described previously.^11^ Blocking of the membranes was performed with either casein blocking buffer (Sigma-Aldrich, Poole, UK) or 5% milk in PBS. Primary antibodies were diluted in the appropriate blocking buffer. Membranes were washed with Tris-buffered saline (10 mM Tris-HCl, pH 7.4, 150 mM NaCl) containing 0.05% (v/v) Tween 20 and incubated with species-specific fluorescent dye–conjugated secondary antibodies diluted PBS. Membranes were washed and analysed using the Odyssey infrared (IR) imaging system (LI-COR Biosciences, Cambridge, UK).

**PCR**

Isolated DNA was amplified using the GoTaq Polymerase Kit (Promega) according to the supplier instructions. Specific primers were used to amplify the Podocin-Cre (fw: 5’-GGATCATCAGCTACACCAGAGACG-3’, rv: 5’-CGCAGAACCTGAAGATGTTCGCGA-3’) and the floxed vinculin (fw: 5’-CCTGCGCGGGATTACCTCATTGAC-3’, rv: 5’-TGCTCACCTGGCCCAAGATTCTTT-3’) alleles. DNA fragments were analysed by agarose gel electrophoresis.

**Quantification of number of foot processes from electron micrographs**

For quantitative ultrastructural analysis of the glomerulus by transmission electron microscopy, the number of podocyte foot processes present in each micrograph was divided by the total length of GBM regions in each image to determine the mean density of podocyte foot processes.^3^

**Immunofluorescence**

Podocytes were seeded on coverslips coated with collagen IV (5 μg/ml) and fixed with 4% paraformaldehyde. Membranes were permeabilised using 0.1% or 0.5% Triton-X. Unspecific protein binding was blocked using 3% BSA for one hour. For kidney tissues, mouse or human kidney renal biopsy cryosections were subjected to antigen retrieval at 95°C for 10 minutes in Retrievagen A solution (pH 6.0) (BD Biosciences), blocked as above, and incubated with the appropriate primary antibodies overnight at 4°C followed by incubation with Alexa Fluor 488 and/or 594–conjugated secondary antibodies at RT for 1 hour. Actin cytoskeleton was stained using phalloidin-647 (Life Technologies, Waltham, USA). Slides were mounted using slow fade mountant containing 4,6-diamidino-2-phenyllinodole (DAPI; Life technologies). Images were acquired with the Nikon Eclipse Ti laser scanning confocal microscope with CSU-W1 camera (Andor technology) using a ×63 Plan Apo (NA = 1.4) oil immersion objective for immunofluorescence analysis, and images were processed using NIH ImageJ software. Both Z-line (side views) and Z-stack (front views) images were obtained using the NIS Elements Imaging Software. The 0.4 µm-to 1 µm fixed interval cuts were acquired, and 20-40 images were generated as a gallery ^13^.

The analysis of the distribution of ZO-1 staining on primary podocytes was performed in a double-blinded manner using NIS Elements Imaging Software and NIH ImageJ software as described previously^14^. The background of all images was subtracted and the fluorescence intensities for the entire cell were independently calculated as F_total_, and the fluorescence intensities of cytosolic region of the same cell was also calculated as F. The ratio of F/ F_total_ podocytes isolated from *Pod-Vcl* KO or control littermate mice treated with LPS or PS was normalized to the F/ F_total_ of vehicle-treated *Pod-Vcl* KO or control littermate podocytes. Three independent experiments were performed.

The analysis of colocalization of nephrin and ZO1 on human FSGS kidney biopsy samples and healthy control human kidney biopsy samples was determined using a JACoP plug-in developed for Image J image analysis software, pearson’s colocalization coefficient (R) was calculated for pixels with intensities above background in a glomerulus from two color images stained with anti-nephrin antibody and anti-ZO1 antibody ^14-16^.

**Ratiometric Imaging Analysis**

Images were acquired on a Delta Vision Core (Applied Precision) restoration microscope using a 60x/ 1.42 Plan Apo objective and the Sedat filter set (Chroma 89000). The images were collected using a Coolsnap HQ2 (Photometrics) camera and analysed using FIJI-ImageJ software. To analyse FA size, background was subtracted using a rolling ball. Cells were co-immunostained with nephrin antibody (GP) to identify podocytes, and pY397-FAK (Rb) and paxillin (Mus) antibodies and images were acquired using the same exposure time between channels. FAs (20 – 25 per cell) were manually selected for analysis by drawing regions of interest (ROIs). The same ROIs were used to measure the integrated density of the FAs in both channels. A ratio of the integrated density between both channels was calculated.

**Immunoprecipitation**

Co-immunoprecipitation (Co-IP) and western blotting form isolated primary podocytes from wild type mice kidney were carried out as described before^17, 18^. Briefly, primary podocyte lysates were prepared in lysis buffer with 1% Triton X-100 containing protease inhibitors. Following centrifugation (10000g and 4 °C for 15 mins) to remove debris, supernatants were precleared with protein A/G plus -agarose beads (Santa Cruz) at 4 °C for 1 hour, then incubated overnight at 4 °C with 30 µl of agarose beads and anti-vinculin monoclonal antibody. Precipitations were washed five times in lysis buffer to remove the unbound proteins, and dissolved in Laemmli sample buffer for western blot analysis.

**References**

1. Yamazaki Y, Umeda K, Wada M*, et al.* ZO-1- and ZO-2-dependent integration of myosin-2 to epithelial zonula adherens. *Molecular biology of the cell* 2008; **19:** 3801-3811.

2. Harita Y, Kurihara H, Kosako H*, et al.* Phosphorylation of Nephrin Triggers Ca2+ Signaling by Recruitment and Activation of Phospholipase C-{gamma}1. *The Journal of biological chemistry* 2009; **284:** 8951-8962.

3. Tian X, Kim JJ, Monkley SM*, et al.* Podocyte-associated talin1 is critical for glomerular filtration barrier maintenance. *The Journal of clinical investigation* 2014; **124:** 1098-1113.

4. Ma H, Togawa A, Soda K*, et al.* Inhibition of podocyte FAK protects against proteinuria and foot process effacement. *Journal of the American Society of Nephrology : JASN* 2010; **21:** 1145-1156.

5. Saleem MA, O'Hare MJ, Reiser J*, et al.* A conditionally immortalized human podocyte cell line demonstrating nephrin and podocin expression. *J Am Soc Nephrol* 2002; **13:** 630-638.

6. Wegener J, Keese CR, Giaever I. Electric cell-substrate impedance sensing (ECIS) as a noninvasive means to monitor the kinetics of cell spreading to artificial surfaces. *Exp Cell Res* 2000; **259:** 158-166.

7. Arif E, Wagner MC, Johnstone DB*, et al.* Motor protein Myo1c is a podocyte protein that facilitates the transport of slit diaphragm protein Neph1 to the podocyte membrane. *Mol Cell Biol* 2011; **31:** 2134-2150.

8. Sheth P, Delos Santos N, Seth A*, et al.* Lipopolysaccharide disrupts tight junctions in cholangiocyte monolayers by a c-Src-, TLR4-, and LBP-dependent mechanism. *Am J Physiol Gastrointest Liver Physiol* 2007; **293:** G308-318.

9. Sun H, Schlondorff J, Higgs HN*, et al.* Inverted formin 2 regulates actin dynamics by antagonizing Rho/diaphanous-related formin signaling. *J Am Soc Nephrol* 2013; **24:** 917-929.

10. Wagner MC, Rhodes G, Wang E*, et al.* Ischemic injury to kidney induces glomerular podocyte effacement and dissociation of slit diaphragm proteins Neph1 and ZO-1. *J Biol Chem* 2008; **283:** 35579-35589.

11. Lennon R, Byron A, Humphries JD*, et al.* Global Analysis Reveals the Complexity of the Human Glomerular Extracellular Matrix. *J Am Soc Nephrol* 2014.

12. Randles MJ, Woolf AS, Huang JL*, et al.* Genetic Background is a Key Determinant of Glomerular Extracellular Matrix Composition and Organization. *J Am Soc Nephrol* 2015; **26:** 3021-3034.

13. Li HC, Kucher V, Li EY*, et al.* The role of aspartic acid residues 405 and 416 of the kidney isotype of sodium-bicarbonate cotransporter 1 in its targeting to the plasma membrane. *Am J Physiol Cell Physiol* 2012; **302:** C1713-1730.

14. Makarenko VV, Usatyuk PV, Yuan G*, et al.* Intermittent hypoxia-induced endothelial barrier dysfunction requires ROS-dependent MAP kinase activation. *Am J Physiol Cell Physiol* 2014; **306:** C745-752.

15. Zerbe KJ. Through the storm: psychoanalytic theory in the psychotherapy of the anxiety disorders. *Bull Menninger Clin* 1990; **54:** 171-183.

16. Parker DJ, Iyer A, Shah S*, et al.* A new mitochondrial pool of cyclin E, regulated by Drp1, is linked to cell-density-dependent cell proliferation. *J Cell Sci* 2015; **128:** 4171-4182.

17. Srichai MB, Konieczkowski M, Padiyar A*, et al.* A WT1 co-regulator controls podocyte phenotype by shuttling between adhesion structures and nucleus. *J Biol Chem* 2004; **279:** 14398-14408.

18. Shono A, Tsukaguchi H, Yaoita E*, et al.* Podocin participates in the assembly of tight junctions between foot processes in nephrotic podocytes. *J Am Soc Nephrol* 2007; **18:** 2525-2533.
